# Clinical Profile of Hospitalized Varicella Cases From Pediatric Emergency Consultations: A Retrospective Cohort Study

**DOI:** 10.7759/cureus.64798

**Published:** 2024-07-18

**Authors:** Hasnae El Haddadi, Amal Hamami, Anane Sara, Aziza Elouali, Ayad Ghanam, Abdeladim Babakhouya, Maria Rkain

**Affiliations:** 1 Department of Pediatrics, Mohammed VI University Hospital, Oujda, MAR; 2 Faculty of Medicine and Pharmacy, Mohammed I University, Oujda, MAR; 3 Department of Pediatrics, Centre Hospitalier Universitaire Mohammed VI, Oujda, MAR; 4 Department of Pediatric Gastroenterology, Centre Hospitalier Universitaire Mohammed VI, Oujda, MAR

**Keywords:** immunization, vaccination programs, rate of hospitalization, ‏acyclovir, varicella-zoster virus

## Abstract

Background: Varicella is a very common childhood infectious disease. It is generally benign, but it can lead to fatal complications. Our study aimed to describe the clinical and therapeutic profile of varicella based on consultations in the pediatric emergency department, to determine the incidence of hospitalized varicella cases in the pediatric department for complementary management, and to specify the incidence of varicella complications in hospitalized patients.

Materials and methods: We conducted a retrospective descriptive cohort study over 12 months. It took place in the pediatrics and pediatric emergency departments of the Mother-Child Hospital of the Mohammed VI University Hospital, Mohammed I University, in Oujda, Morocco.

Results: We collected 120 cases of varicella. The mean age of patients was 4.5 years. The most common age range was 4-6 years (69%). Males predominated. The reason for consulting the pediatric emergency department was a febrile rash in 65% of cases. Treatment in pediatric emergencies was mostly symptomatic. Antibiotic treatment for superinfection of lesions was used in 11% of cases. The number of hospitalizations due to complicated and/or severe varicella was 17 cases. The median age was 6.3 years. Most of the children (82%) were immunodeficient and 18% were immunocompetent. Sixteen patients had underlying risk factors. Infectious skin and soft tissue complications were noted in most hospitalized patients (47%). They were mainly presented by cutaneous reinfections with alteration of general health (41%). Neurological complications ranked second (23%). The majority were febrile convulsions (17%). One case of bronchopulmonary complication was noted. No hematological, digestive, renal, or cardiac complications were noted. Intravenous antiviral treatment was used in 88% of hospitalized cases. The drug of choice was acyclovir. Antibiotic therapy was used in 53% of cases. No patient received corticosteroid therapy. The median length of hospitalization for our patients was 14 days. The evolution was favorable in 100% of cases.

Conclusion: Varicella remains a benign disease in children, rarely leading to hospitalization. However, complications may develop in cases of comorbidity or children with risk factors. The introduction of the varicella vaccine into the national immunization program could considerably reduce the number of children hospitalized in the near future.

## Introduction

Varicella (chickenpox) is an infectious, eruptive, almost ubiquitous disease of childhood. It is caused by the varicella-zoster virus (VZV), also known as the chickenpox virus [1]. A member of the herpes virus family, it causes both chickenpox, a common childhood illness characterized by a rash, and shingles, a reactivation of the virus in people who have already had chickenpox. VZV is a double-stranded deoxyribonucleic acid (DNA) virus with a virulent envelope. Its reservoir is strictly human. There is only one VZV serotype, but at least seven different classes have been identified [2].

Chickenpox is a highly contagious disease [1]. The virus is transmitted mainly by the respiratory route via droplets and secretions, from the liquid contained in the vesicles, or indirectly via contaminated objects. The attack rate in a susceptible subject is 86.6% after intrafamilial contact and 10-35% after less intimate contact in a community [3]. Varicella evolves through seasonal epidemics that occur in late winter and spring. It is essentially a childhood disease, with 50% of children contracting the infection before the age of five and 90% before the age of 12 [2].

In immunocompetent children, varicella is considered a benign disease, whereas it can lead to fatal complications in the immunocompromised. However, it is the cause of significant morbidity and hospitalization costs, due to its frequency and complications, which mainly affect immunocompromised patients and children with risk factors [4].

Varicella can be prevented by vaccination [5]. The varicella vaccine was produced in M. Takahashio's laboratory at Osaka University in the 1970s. However, it was not approved for general use until the 1990s. The marketing authorization reserves the vaccine for healthy individuals over one year of age, in a single dose for children aged 1-2, and in two doses separated by 4-8 weeks for children over 13. The vaccine achieves seroconversion in almost 100% of cases, after one dose in infants and children and two doses in adolescents [6].

This retrospective descriptive cohort study aims to describe the clinical and therapeutic profile of varicella based on consultations in the pediatric emergency department, to determine the incidence of varicella cases hospitalized in the pediatric department for complementary management, to assess their vaccination status, and to specify the incidence of varicella complications in hospitalized patients.

## Materials and methods

Study design

We conducted a retrospective descriptive cohort study over 12 months (January 2023 to December 2023). It took place in the pediatrics and pediatric emergency departments of the Mother-Child Hospital of the Mohammed VI University Hospital, Mohammed I University, in Oujda, Morocco. The study combined an observational approach based on consultation registers in the pediatric emergency department and archived files in the pediatric department, with a descriptive approach based on results obtained using statistical software. 

Study population

During the study period, we collected 120 cases of varicella, 17 of them hospitalized in the pediatric department. The inclusion criteria used in our study were as follows: children under 16 years of age who consulted the pediatric emergency department for the management of varicella and were treated on an ambulatory basis or who needed to be hospitalized in the pediatric department for complementary management. We excluded patients over 16 years of age or consulting for a febrile rash other than varicella.

Data collection and analysis

Data were obtained from pediatric emergency department consultation registers and archived records of patients hospitalized in the pediatric ward. An Excel file (Microsoft® Corp., Redmond, Washington, United States) was created to collect patients' anamnestic, vaccination, clinical, and therapeutic data. Categorical variables were expressed as frequencies, while numerical variables (age, etc.) were presented as means.

## Results

**Figure 1:**
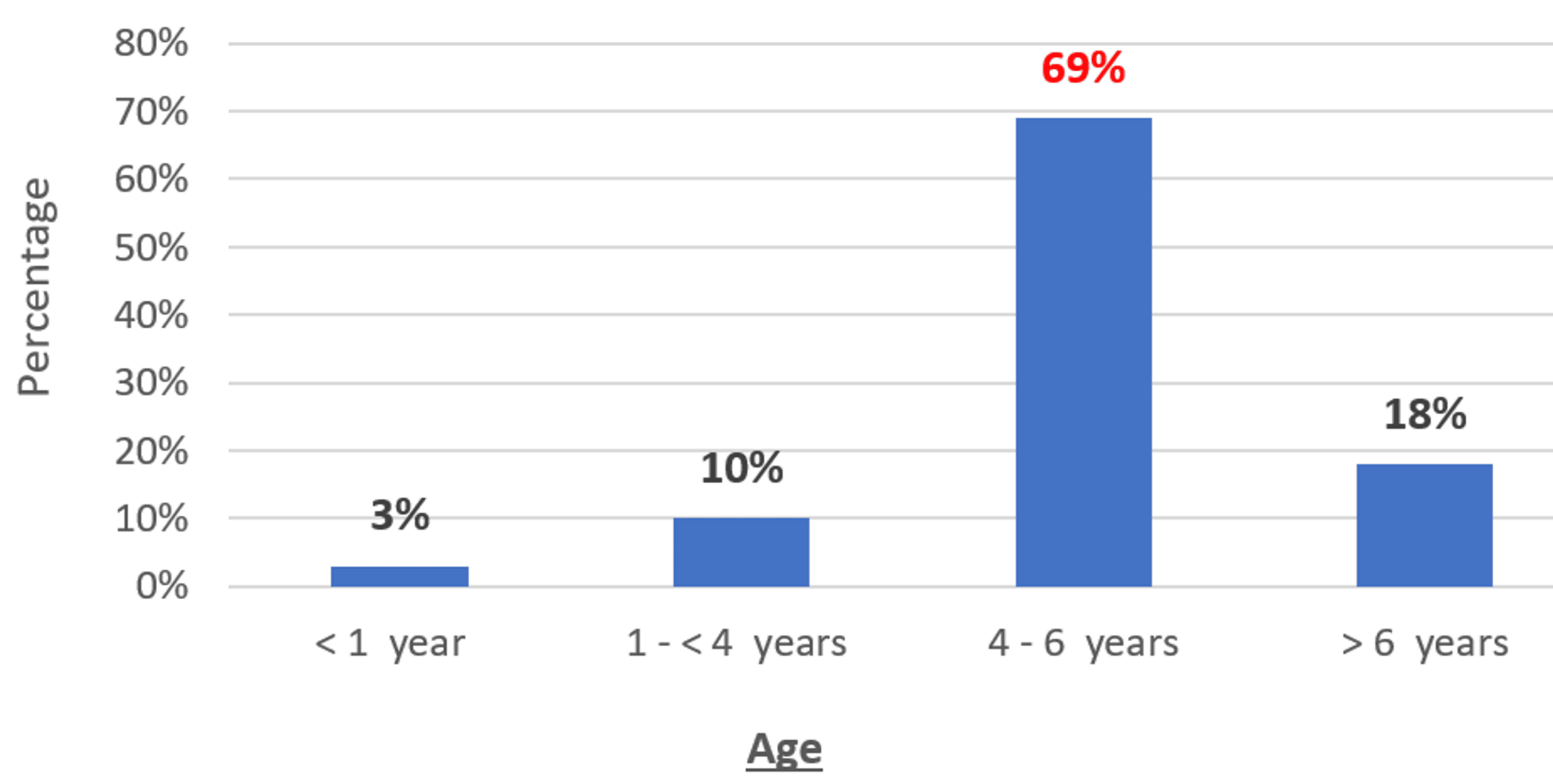
Age distribution of patients (n=120 cases)

**Figure 2:**
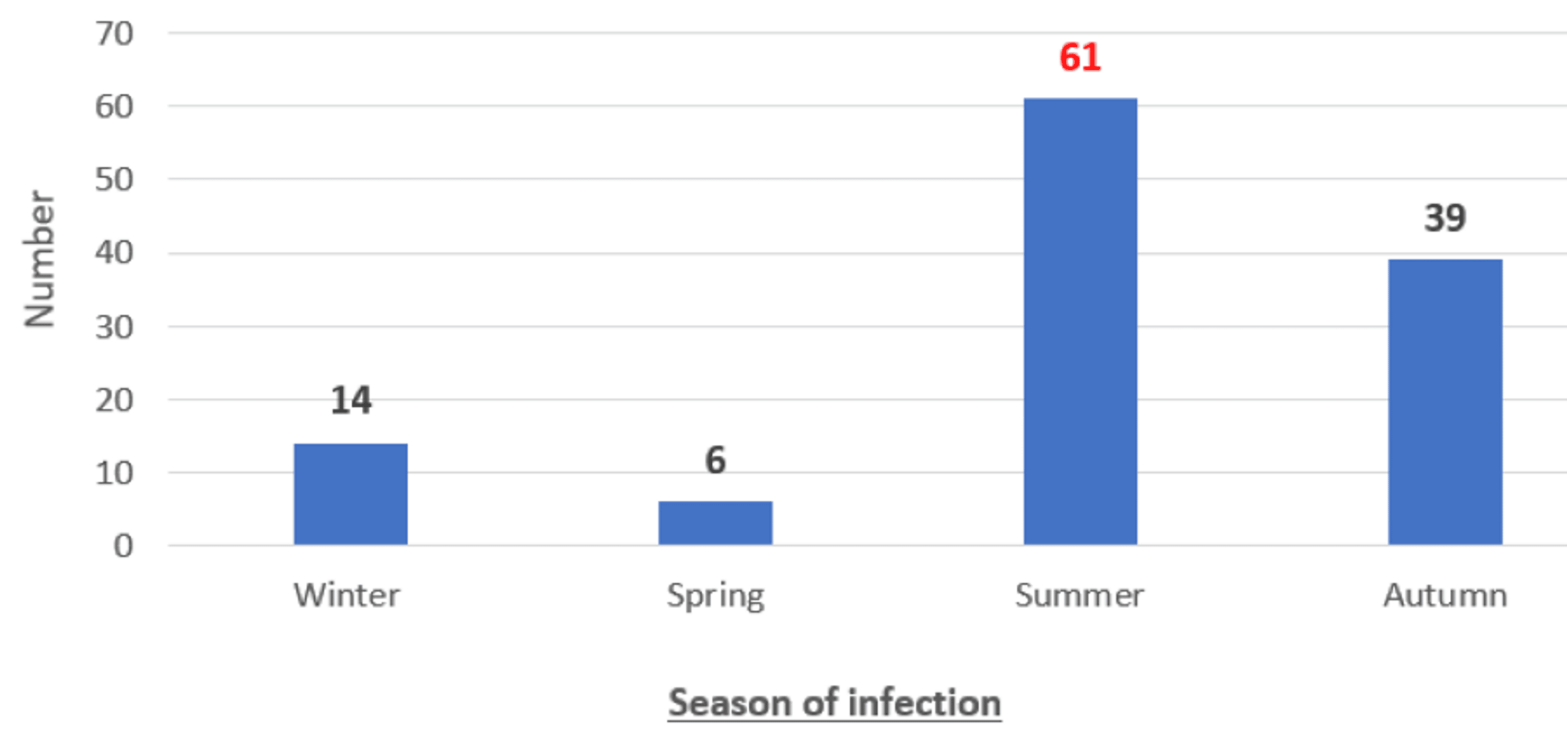
Distribution of patients by season of infection (n=120 cases)

**Figure 3:**
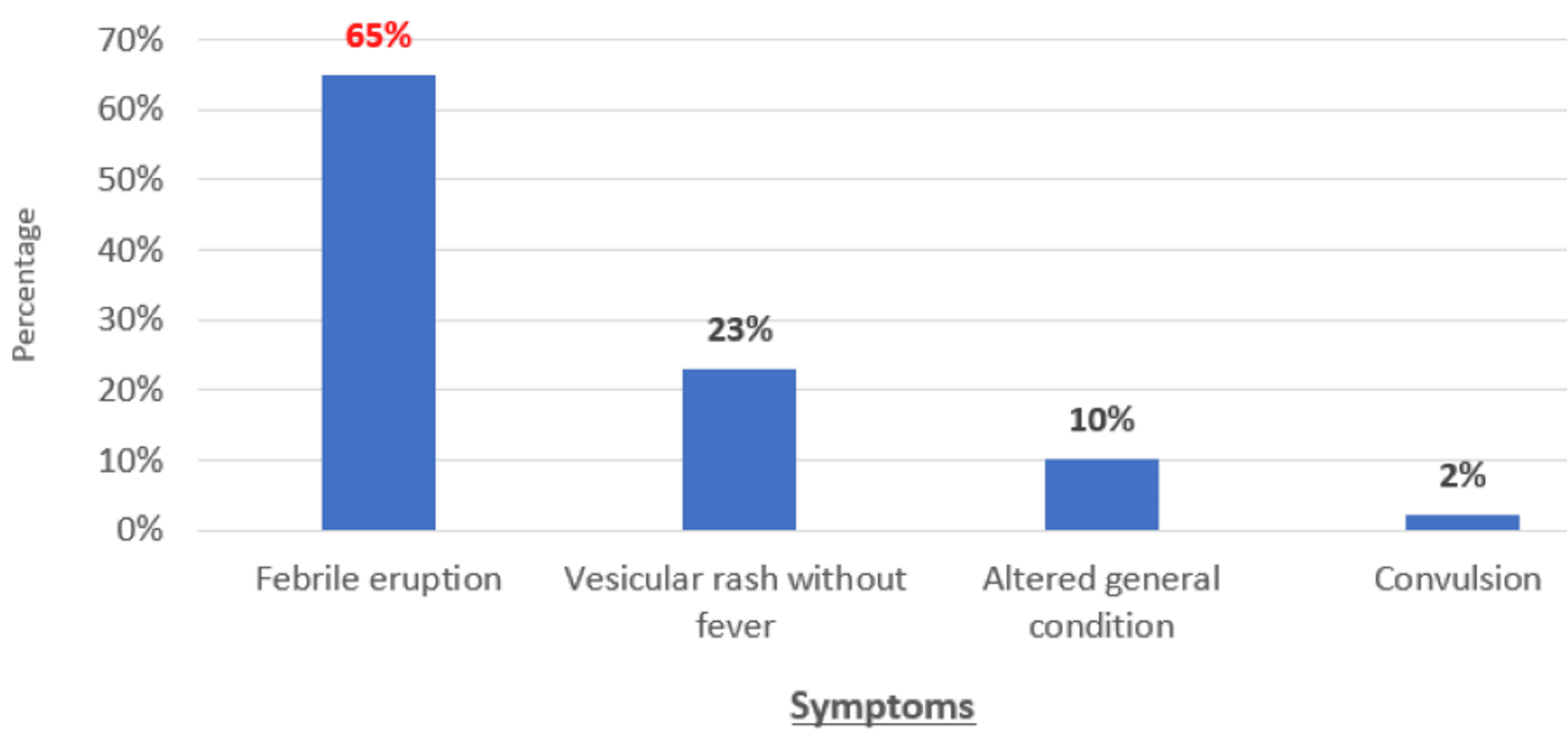
Distribution of patients by reason for consultation in the pediatric emergency department (n=120 cases)

The mean age of patients was 4.5 years. The most common age range was 4-6 years (n=82; 69%). Males predominated (n=86; 72%) (Figure 1). The most common season of infection was summer (n=61; 51%) (Figure 2). Infection was most often familial. All our patients were vaccinated according to the national immunization program (n=120; 100%), but none had received the varicella vaccine. The reason for consulting the pediatric emergency department was as follows: a febrile rash (n=78; 65%), a vesicular rash without fever (n=28; 23%), an altered general health (n=12; 10%), and a convulsion (n=2; 2%) (Figure 3).

Treatment in pediatric emergencies was mostly symptomatic, based on antipyretics, antihistamines, and local antiseptics. Antibiotic treatment for superinfection of lesions was used in 11% of cases (n=13). Fifteen percent of cases required hospitalization in the pediatric ward for complementary management and possible intravenous antiviral treatment (n=17; 15%).

Characteristics of patients hospitalized in the pediatric department

The number of hospitalizations due to complicated and/or severe varicella recorded during the study period was 17 cases. The median age was 6.3 years, with extremes of eight months to 15 years. Seventy-five percent of the children were over three years of age, and two patients were under one year. A predominance of males was noted, with a sex ratio of 1.8. Most of the children were immunodeficient (n=14; 82%) and 18% immunocompetent. 

**Figure 4:**
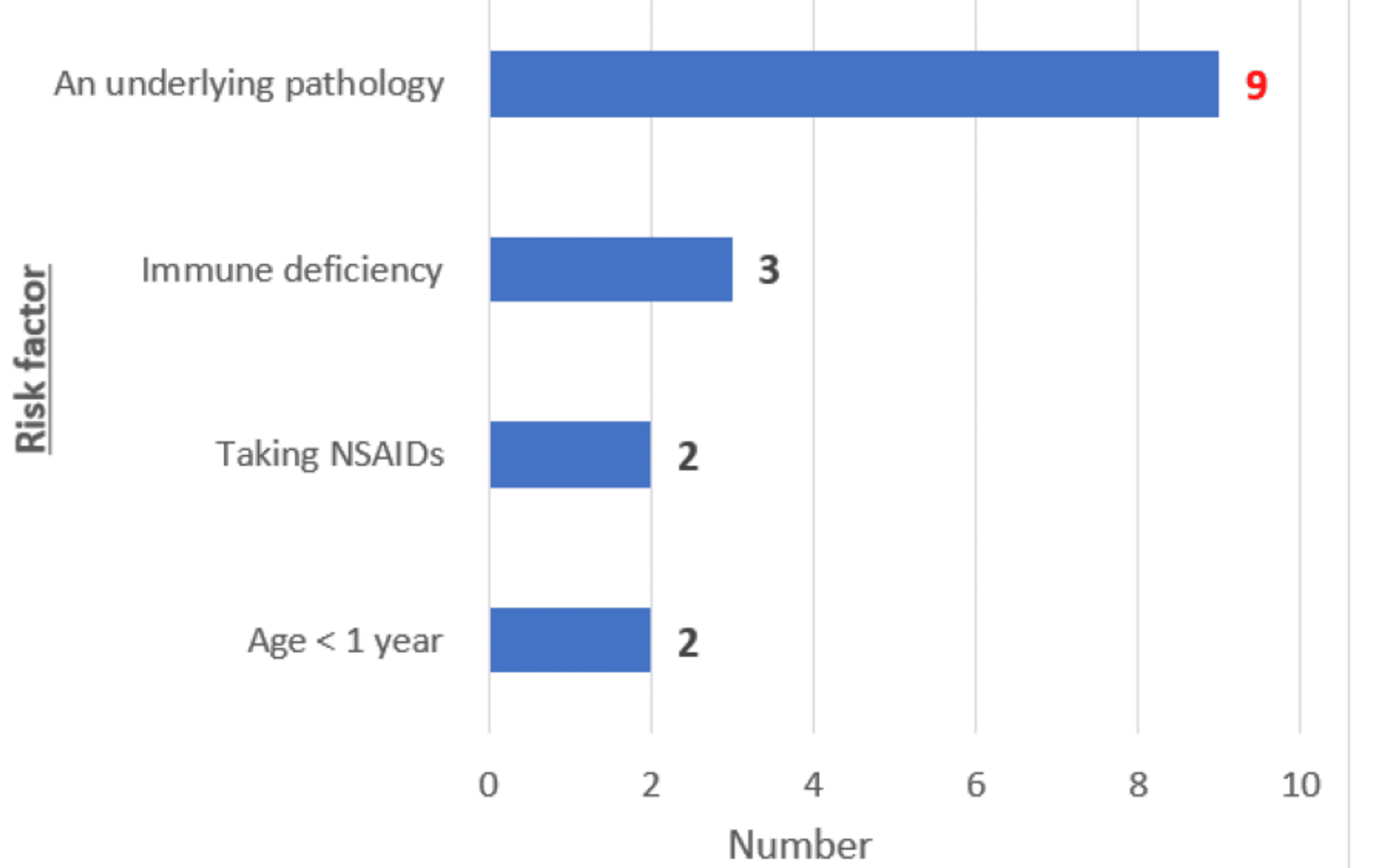
Distribution of patients according to risk factors (n=16) NSAIDs: non-steroidal anti-inflammatory drugs

Sixteen patients had underlying risk factors: age less than one year (n=2; 12%), congenital immune deficiency (n=3; 17%), non-steroidal anti-inflammatory drugs (n=2; 12%), presence of an adjacent pathology such as acute leukemia (n=5; 29%), bone marrow aplasia (n=2; 12%), non-Hodgkin's lymphoma (n=1; 6%), and juvenile idiopathic arthritis on biotherapy (n=1; 6%) (Figure 4). 

Infectious skin and soft tissue complications were noted in most hospitalized patients (n=8; 47%). They were mainly presented by cutaneous reinfections with alteration of general health (n=7; 41%) and impetigo (n=2; 12%). *Staphylococcus aureus* was isolated in two cases and *Streptococcus pyogenes* in one. There were no cases of cellulitis, dermo-hypodermatitis, lymphangitis, abscesses, or ulceronecrotic lesions. Neurological complications ranked second (n=4; 23%). The majority were febrile convulsions (n=3; 17%). Cerebellar ataxia secondary to cerebellitis was the second most frequent neurological complication (n=1; 6%). Cerebrospinal fluid (CSF) examinations of patients with neurological complications were without abnormalities. One case of bronchopulmonary complication was noted, in the diffuse interstitial lung disease type. No hematological, digestive, renal, or cardiac complications were noted.

Intravenous antiviral treatment was used in 88% of hospitalized cases, given the underlying condition and the severity of the clinical symptoms. The drug of choice was acyclovir 10 mg/kg/8h for all patients for 7-14 days. In the case of skin superinfections, an antibiotic treatment was used in 53% of cases, based on amoxicillin-clavulanic acid, with an average duration of seven days. No patient received corticosteroid therapy. The median length of hospitalization for our patients was 14 days. The evolution was favorable in 100% of cases. No cases of death or sequelae were noted.

## Discussion

We retrospectively studied cases of varicella treated in the pediatric emergency department or hospitalized in the pediatric department. Among 15,623 consultations recorded over the study period, the incidence of admissions of varicella cases to the pediatric emergency department was 120 cases/year. This is the same number reported by Ziebold et al. in Germany (119 cases/year) [7] and lower than that reported in several other countries, including Switzerland (420 cases/year) [8], the United Kingdom and Ireland (288 cases/year) [9], and Belgium (552 cases/year) [10]. Due to major differences in study design and health systems, it was difficult to compare the incidence and epidemiology of varicella in Morocco with other countries, but most studies have reported an increase in the incidence of hospitalized cases of varicella in the pediatric population worldwide, with a change in the age distribution of children hospitalized for complicated varicella [11,12].

Several studies have been carried out to evaluate the complications of varicella, and several risk factors for severe or complicated varicella have been identified, with age being the most important factor [2]. Children under five years of age are at high risk of complications, particularly infectious ones, and mortality in infants under one year of age is four times higher than in older children [13]. Other risk factors have been demonstrated, such as the use of corticosteroids and non-steroidal anti-inflammatory drugs, underlying eczema, asthma or other diseases, intra-familial contamination, and immunodeficiency [2]. In our study, the most common risk factor was acquired or congenital immunodeficiency.

Bacterial superinfections of the skin and soft tissues remain the most frequent complications reported in the literature, the most serious being necrotizing fasciitis. The germs most frequently implicated are *Staphylococcus aureus* and *Streptococcus pyogenes* [1]. The study by Wen et al. showed that among 140 hospitalized children, skin infections remained the most frequent complication, accounting for 75% of cases [14]. Neurological complications come in second place and classically represent almost 1/3 of the causes of hospitalization in the literature, with results identical to those reported in our study. They include cerebellitis, meningoencephalitis, Reye's syndrome, stroke, and other less rare neurological complications such as transverse myelitis, Guillain-Barré syndrome, and optic neuritis [3]. In Germany, neurological complications account for most of the complications reported by Ziebold et al. (61.3%) [7]. Unlike in adults, pneumopathy is a rare complication in children with no particular risk factors, except in infants under six months, in whom it is the predominant cause of mortality [15]. A distinction should be made between bacterial superinfections, in the form of pneumopathy or pleuro-pneumopathy, most often caused by pneumococcus, hemolytic streptococcus, or staphylococcus, which account for the vast majority of cases, especially in previously healthy children, and interstitial pneumopathy directly caused by the varicella virus, most often occurring in immunocompromised individuals [2,15].

Safe and effective live attenuated varicella vaccines were developed in the 1970s. As a result, universal varicella vaccination has been implemented for over 10 years in many countries such as the United States, Canada, Australia, and Germany, showing conclusive efficacy results [10]. According to recent American and European studies, varicella vaccination has led to a reduction of at least 88% in varicella incidence, mortality, complicated cases, and hospitalizations. Although there was a slight shift in peak incidence towards older children, the impact of vaccination was significant in all age groups [16,17].

Varicella vaccine is not yet included in Morocco's national vaccination program. However, it is available in the private sector but is not compulsory and can be administered to children from the age of 12 months, which means that vaccination coverage may be low compared with countries where the vaccine is routinely administered. This can lead to greater virus circulation and occasional epidemics [13].

Limitations of this study include the retrospective design of the chart analysis, with only information recorded in patient dossiers being entered, which may have led to an underestimation of the results. We also assumed that, due to the high rate of generalized health coverage and the implementation of the compulsory health insurance system in Morocco, complicated cases of varicella may be managed in the private sector, which may have underestimated the complicated cases hospitalized in our department. Despite these limitations, however, this is the first study to report the frequency of consultations for varicella in pediatric emergency departments and the prevalence of hospitalization of complicated cases in the pediatric department of the Oujda University Hospital, Morocco.

## Conclusions

Varicella remains a benign disease in children, rarely leading to hospitalization. However, complications may develop in cases of comorbidity or children with risk factors. These could aggravate the prognosis in both children and adults, even in a referral healthcare setting. Treatment of varicella is generally symptomatic, aimed at relieving symptoms and preventing complications. In complicated cases, intravenous acyclovir should be started. The introduction of the varicella vaccine into the national immunization program could considerably reduce the number of children hospitalized in the near future. 
